# Investigation of the structural, functional, and digestive properties of beta-casein from cow, goat, and donkey milk

**DOI:** 10.1016/j.fochx.2025.102543

**Published:** 2025-05-11

**Authors:** Guiqin Liu, Ning Wang, Xue Chen, Yaqian Jin, Junnan Wan, Yanhao Zhao, Yiting Zhao, Cunfang Wang

**Affiliations:** a College of Agriculture and Biology, Liaocheng University, Shandong Engineering Technology Research Center for Efficient Breeding and Ecological Feeding of BlackDonkey, Shandong Donkey Industry Technology Collaborative Innovation Center, Liaocheng, China; b College of Food Science and Engineering, Qilu University of Technology (Shandong Academy of Sciences), Jinan 250353, China

**Keywords:** Beta-casein, Different milk sources, Physical–chemical properties, Antioxidant, Digestive properties

## Abstract

This study aimed to investigate the genotypes, structures, physicochemical properties, in vitro antioxidants, and digestive properties of isolated goat, donkey, and cow β-casein (β-CN). The β-CN genotypes of goats and donkeys are not cow type A1 β-CN, avoiding the unfavorable effects on intestinal digestion of BCM-7, a peptide fragment produced by the digestion of cow type A1 β-CN from bovine milk. Goat and donkey β-CN exhibited a smaller particle size, a more stable solution system, a looser secondary structure, and better solubility than cow β-CN. The microstructural findings from simulations of in vitro digestion in infants revealed that goat β-CN exhibited superior digestibility, characterized by reduced flocculation and a more loosely organized protein structure during the gastric digestion phase. Furthermore, the degree of hydrolysis indicated that both goat and donkey β-CN were significantly more readily digested compared to cow β-CN. Moreover, donkey β-CN gastrointestinal digests possessed significant anti-free radical activity.

## Highlights

• Exploring the Benefits of goat and donkey β-CN as a formula for infant.
• Comparison of β-CN structure and function in cow, goat and donkey.
• Goat and donkey β-CN avoid the negative effects of cow A1 β-CN.
• β-CN from goats and donkeys is easily digestible in infant digestion.
• Donkey β-CN digests had good antioxidant properties.

## 1 Introduction

Breast milk is the optimal food source for infants, and the World Health Organization recommends exclusive breastfeeding for the first six months of life. The ratio of casein (CN) to whey protein in mature breast milk is generally 40:60, with β-casein (β-CN) as the most abundant protein in CN. To address the growth and developmental requirements of infants, the nutritional formulation of infant formula is designed to closely resemble that of breast milk. In recent years, there has been a heightened awareness regarding the safety, digestibility and gastrointestinal health associated with infant formulas. Consequently, the incorporation of various sources of β-CN in infant formulas as a substitute for the β-CN found in breast milk has attracted much attention.

Cow β-CN contains approximately 30 % of the total casein. Analysis of the percentage of β-casein variants in the dairy population reveals that both A1 and A2 essentially range from 37 % to 55 %. The digestion of type A1 beta-casein produces the peptide fragment BCM-7, which may be associated with lactose intolerance, gastrointestinal distress, and type 1 diabetes (Vigolo et al., 2022). Type A2 beta-casein has more stable physicochemical properties and is more beneficial to the gastrointestinal health of different populations compared to type A1 beta-casein. Previous research has determined the β-casein subtype of goat milk as A2 (Oliveira et al., 2023), yet that of donkey milk remains unknown.

The structural and functional properties, digestion, and absorption vary between different β-CN sources. Camel β-CN is observed to exhibit better antioxidant, antifungal, and emulsifying activities than that of cow β-CN (Lajnaf et al., 2021). β-CN from different milk sources have a similar self-assembly behavior (Perinelli et al., 2019). Goat milk has a high digestibility and beta-casein percentage, low allergenicity, and a wide range of functional properties and health-promoting benefits. β-casein contains high proportion of beneficial amino acid residues (Pro, Tyr, Trp, and Phe) and has an inhibitory effect on Angiotensin converting enzyme (ACE) (Y. Wu et al., 2023). Donkey milk has antibacterial, antiviral, immunomodulatory, anti-inflammatory, antioxidant, and anti-tumor activities, among other biological activities. Moreover, donkey milk has the highest content of β-casein among casein, as well as low immunogenic potential. Scholars have demonstrated that donkey β-CN could not be recognized by the cow β-CN allergy antibody (Perinelli et al., 2019). Goat and donkey milk have attracted an increasing amount of attention due to their lower allergenicity, strong bioactivity, and protein composition that is in favor of human milk. Although several studies have proposed the chemical–physical properties and applications of β-CN, they all focus on proteins of bovine origin (Ye et al., 2019), while research on the physicochemical properties and digestive characteristics of β-CN in goat and donkey milk is limited. The study of the structure and function of beta-casein across various milk sources can help optimize dairy products, especially infant formulas, to be closer to breast milk, reduce the risk of allergy and enhance the nutritional value. Analyzing the digestive properties of beta-casein can provide insights into the absorption efficiency associated with different variants of this protein. Meanwhile, the digestive properties of beta-casein directly affect the absorption efficiency of minerals such as calcium and iron in infants. Additionally, the physicochemical properties of beta-casein play a significant role in the processing of dairy products.

As a major casein in breast milk, β-CN plays a key role in the growth and development of infants and young children. This study aims to: i) determine the physicochemical functions and digestive properties of β-CN from goat and donkey milk; ii) optimize the choice of β-CN in infant formula; and iii) improve the application of β-casein from different milk sources in the field of dairy products.

## 2 Materials and methods

### 2.1 Materials

Cow milk samples were obtained from Shandong Xingniu Dairy Co., Ltd. (Jinan, China). Goat milk samples were from the Otter breed goat farm of the Qingdao Animal Husbandry and Veterinary Research Institute (Qingdao, China). Donkey milk samples were obtained from Liaocheng Donge Ejiao Co., Ltd. (Liaocheng, China). The samples were free of acute mastitis and other diseases. The milk was mixed evenly after sampling by the milking machine in the milking parlor and was then transported to the laboratory through a cold chain for subsequent analysis. Pepsin (activity: 3000 U/mg), trypsin (activity: 250 U/mg) and bile salt were purchased from Macklin Co., Ltd. (Shanghai, China). Ultrapure water was produced from Direct-Q® 5 UV, Millipore SAS (Massachusetts, USA). All chemicals and reagents were of analytical grade, unless otherwise noted.

### 2.2 Fabrication of β-casein

The extraction of β-casein was conducted following the method developed by Chen et al. (2024). Three milk samples were subjected to centrifugation at 4 °C to remove the upper layer of milk fat. Subsequently, the samples were alkalized to a pH of 11.0 using NaOH (1 M) at 37 °C. CaCl_2_ solution was added to obtain the final Ca^2+^ concentration of 65 mM and the pH was adjusted to 7.0 with HCl (1 M). The samples were centrifuged to facilitate the precipitation of β-casein and α_s_-casein. A portion of the precipitates was freeze-dried to obtain casein powder from the cow, goat, and donkey milk, denoted as CCN, GCN, and DCN respectively. The remaining precipitates were redissolved by the addition of ultrapure water, followed by cooling at 2 °C for 24 h. The mixture was then centrifuged to isolate the supernatant. The pH of the supernatant was adjusted to 4.5 (all three beta-caseins precipitated at this pH) and heated to 35 °C to precipitate β-CN. The freeze-dried β-casein derived from cow, goat, and donkey milk was designated as CBCN, GBCN, and DBCN respectively. Finally, dissolve the freeze-dried samples in phosphate-buffered saline (PBS) buffer (0.1 M, pH 7.2–7.6) to obtain crude protein solutions with a concentration of 5 mg/mL. After determining the protein purity, a precise protein solution at a concentration of 5 mg/mL was prepared for subsequent experiments.

### 2.3 Sodium dodecyl sulfate polyacrylamide gel electrophoresis (SDS-PAGE) and protein content

Equal volumes of sample and buffer (0.05 mol/L Tris-HCl, pH 8.0, containing 0.1 g/L SDS and 100 mL/L glycerol) were mixed and then heated in boiling water for 3 min. Subsequently, SDS-PAGE using 5 % concentrated gel and 12 % separated gel at 130 V for 90 min. The gel was stained with Kaomas Brilliant Blue R-250 and decolorized in 7.5 % acetic acid. The content of the β-casein was determined by Kjeldahl nitrogen determination (Zhang et al., 2022).

### 2.4 Reversed-phase high-performance liquid chromatography

Reversed-phase high-performance liquid chromatography (RP-HPLC) was employed to determine the purity and genotype of β-CN. Reagent I (mixed solution of bis-trimethylol aminomethane 0.1 mol/L, guanidine hydrochloride 6 mol/L, sodium citrate 5.37 mmol/L, dithiothreitol 19.5 mmol/L) and reagent II (guanidine hydrochloride 4.5 mol/L) were prepared with 0.1 % trifluoroacetic acid solution as the solvent. The mobile phases were as follows: A, 0.1 % trifluoroacetic acid aqueous solution; B, pure acetonitrile (chromatographic grade). 4 mg/mL of β-CN was dissolved with reagent I, left for 1 h, and the underlying solution of 300.0 μL was mixed with 900.0 μL of reagent II to obtain a final protein concentration of 1 mg/mL. Gradient elution is employed. The samples were filtered (0.45 μm) and analyzed using a Prominence LC-20 A RP-HPLC system (Shimadzu, Japan) equipped with a C18 column (XBridge® Peptide BEH, 4.6 mm × 250 mm, 3.5 μm).

### 2.5 Particle size, polydispersity index

Based on a previously reported method (D. Z. Liu et al., 2012), the particle size and polydispersity index (PDI) were determined via the dynamic light scattering technique using a Zetasizer Nano-ZS90 device (Malvern Instruments Limited, UK).

### 2.6 Zeta potential

Zeta potential measurements of the samples were performed using laser Doppler velocimetry (Malvern Instruments Limited, UK).

### 2.7 Free sulfhydryl content

The free sulfhydryl group was determined by the DTNB method described in Ellman (2022).

### 2.8 Secondary structure of β-casein

Fourier Transform Infrared (FTIR) spectra of the samples were collected with a Spectrum 100 FTIR spectrometer (PerkinElmer, UK), following the methodology outlined by F. Liu et al. (2017). FTIR spectra were obtained after 64 scans in the mid-infrared region (4000–400 cm^−1^) and analyzed using Omnic 8.0 Thermo Fisher Scientific Inc., Waltham, MA, USA). The amide I band is in the region of 1700–1600 cm^−1^. Second-derivative analysis (“peak fitting”) of the IR-SD, The secondary structure components of the complexes were quantitatively analyzed.

### 2.9 Turbidity and solubility of β-casein

The turbidity for all samples was measured at 860 nm using an ultraviolet–visible (UV–Vis) spectrophotometer (UV756, Yoke Instrument Co. Shanghai China). The concentration of the β-casein solutions was further diluted with ultrapure water to 2 mg/mL and the pH value was adjusted from 3 to 11. Centrifuged was then performed at 25 °C at 10,000 ×g for 20 min. The total protein content and protein content of the supernatant were determined by the BCA method to calculate the solubility (Y. Liu et al., 2015).

### 2.10 Surface hydrophobic and interface characteristic

The surface hydrophobicity was determined using ANS as a fluorescent probe (Han et al., 2020). The fluorescence intensity of the solution was measured by a fluorescence spectrophotometer (F-2700, HITACHI Co. Japan). The fluorescence intensity and protein concentration curve was fitted by the linear regression method, and its slope was taken as the surface hydrophobicity index (Ho).

The foaming capacity (FC) and foam stability (FS) were determined based on previous research (Meng & Li, 2021). The emulsification activity index (EAI) and emulsification stability index (ESI) were calculated based on previous research (J. Li et al., 2019).

### 2.11 Antioxidation analysis

The concentration of the β-casein solutions was further diluted with ultrapure water to 2 mg/mL. Antioxidant activity was assessed using •OH and ABTS scavenging ability. Hydroxyl radical scavenging activity was quantified at 517 nm by referring to the method of Yuan et al. (2013).

•OH radical scavenging assay was performed with UV–Vis spectroscopy (UV756, Yoke Instrument Co. Shanghai China) at 517 nm according to the method of Yuan et al. (2013), with slight modifications. ABTS radical scavenging activity was determined at 734 nm by referring to the method of B. Wang et al. (2012).

### 2.12 Vitro-simulated infant digestion

Vitro-simulated infant digestion was evaluated following previously described methods, with some modifications. In brief, the sample solution was mixed with simulated gastric fluid (containing pepsin with an activity of 227.5 U/mL) at a final ratio of 1:1 (*v*/v). After the pH was adjusted to 4.0, simulated gastric digestion was performed at 37 °C for 120 min. Digestive samples were collected at 30, 60, 90, and 120 min during gastric digestion and heated at 95 °C for 5 min to inactivate enzymes, denoted as G30, G60, G90, and G120, respectively. After the pH was adjusted to 6.6 at the end of the gastric phase, in vitro enteric digestion was performed at 37 °C for 120 min. Trypsin activity was 8.63 U/mL, and digestion samples were collected at 15, 30, 60, and 120 min during gastrointestinal digestion and heated at 95 °C for 5 min to inactivate enzymes, denoted as I15, I30, I60, and I120.

### 2.13 Hydrolysis degree

The hydrolysis degree (DH) represents the percentage of peptide bond cleavage in protein hydrolysates. It was evaluated according to the approach of Jiang et al. (2024). A spectrophotometer (UV756, Yoke Instrument Co. Shanghai China) was employed to record the absorbance at 340 nm.

### 2.14 Microstructure

The microstructure changes of the β-casein samples during digestion were observed by a confocal laser scanning microscope. The protein was labeled with 0.01 % (*w*/*v*) solid green. A total of 20 uL solid green solution was added to 2 mL sample solution, mixed well, and reacted for 20 min away from light. Following this, 10 uL of the liquid sample was taken, placed on a slide, and covered. For measurements, the excitation wavelength was set to 633 nm.

### 2.15 Statistical analysis

All operations were conducted in triplicate and the data were expressed as the average ± standard deviation of the values of three sample replicates. Statistical analysis was performed in SPSS v. 22 (IBM Corp., New York, USA) with one-way analysis of variance at a significance level of *P* ± 0.05. OriginPro 8.5 (OriginLab, Massachusetts, USA) was used to plot the results.

## 3 Results and discussion

### 3.1 Casein content and genotype speculation

**Fig. 1:**
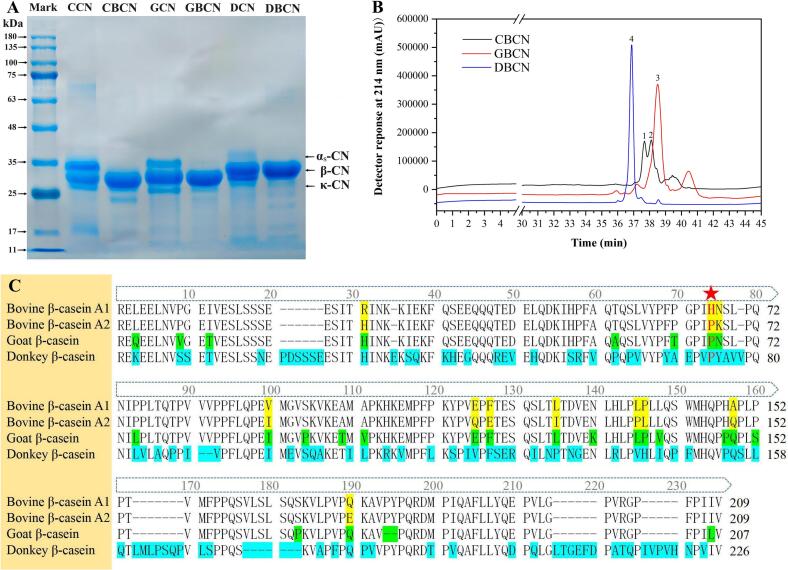
SDS-PAGE (A), reverse high-performance liquid chromatography, and (B) sequence alignment (C).

The SDS-PAGE diagram in Fig. 1A presents the typical bands of different types of casein in cow, goat, and donkey milk. The locations of these bands indicate that the molecular weight and component content of casein from the different milk sources were significantly different. Cow milk casein was mainly composed of α_s_-CN (∼26 kDa) and β-CN (∼24 kDa). The greatest molecular weight of β-CN was determined in donkey milk (∼25.5 kDa), followed by goat milk (∼24.8 kDa), and cow milk (Uniprot database, https://www.uniprot.org). Goat and donkey milk contain a higher proportion of β-CN in the total CN content compared with the CN control group. There were no impurity protein bands—except for the β-CN bands—in the electrophoretic map extracted from the β-CN group. This shows that the adopted extraction method can efficiently separate β-CN from milk. The β-CN protein content was calculated to be 80.52 %, 76.84 % and 98.34 % for cow, goat and donkey, respectively, using Kjeldahl nitrogen determination.

Liquid chromatography analysis (Fig. 1B) revealed that all three β-casein samples displayed prominent peaks at approximately 38 min into the measurements, with no significant extraneous peaks. This implies that the peak retention time for β-casein is around 38 min, suggesting that the samples are predominantly free of heteroprotein, which is consistent with the results obtained from the electropherogram for β-casein. The β-CN purity levels in the samples derived from cow, goat, and donkey milk were found to be 84.44 %, 91.97 %, and 98.85 %, respectively. CBCN exhibited two distinct peaks corresponding to β-casein at 37.679 min and 38.108 min. These peaks are associated with the A1 and A2 variants of β-casein present in milk, with the peak at 37.679 min identified as A1 β-CN and the peak at 38.108 min as A2 β-CN. The peak retention time of β-casein derived from goat milk was recorded at 38.508 min, which is comparable to the A2 variant of β-casein in cow milk. Previous studies have indicated that the β-CN present in goat milk is classified as A2 (Oliveira et al., 2023). In contrast, the peak retention time for β-casein in donkey milk was observed at 36.872 min, which does not correspond to the peak times of the A1 and A2 variants in cow milk β-casein. Thus, the classification of the genotype of β-casein in donkey milk remains unclear. The differentiation between β-casein types A1 and A2 is predicated on the specific amino acid sequence at position 67, where histidine is present in type A1 and proline in type A2. To determine the variant type of β-casein, amino acid sequences from various milk sources were retrieved from the National Center for Biotechnology Information database (https://www.ncbi.nlm.nih.gov). Fig. 1C presents the amino acid sequences of the three β-casein variants. Note that the β-casein derived from goat milk contains proline at position 67, a characteristic that is also present in the β-casein from donkey milk. Thus, the β-casein from donkey milk aligns with the A2 variant type. Furthermore, both GBCN and DBCN do not correspond to the A1 β-CN variant, as indicated by the amino acid sequence at position 67, thereby mitigating the potential adverse effects associated with the A1 variant. Cow A1 β-casein releases BCM-7 (YPFPGPI) in digestion, which may cause digestive discomfort. A2 β-casein releases BCM-7 in digestion at a much lower concentration than A1 β-casein. Because the proline at position 67 is less susceptible to elastase cleavage compared to histidine. Fig. 1C suggests that the proline at position 67 in goat and donkey β-casein may be functionally similar to “A2 β-casein”. In addition, goat and donkey β-caseins differ in amino acid sequence within the cow BCM-7 region. If released, the peptides produced by goat and donkey β-casein in the cow BCM-7 region are YPFTGPI and YPYAEPV, respectively. The release and potential physiological effects of these peptides have yet to be investigated. And these two peptides are not the BCM-7 that causes digestive discomfort.

### 3.2 Particle size, PDI of β-CN

**Fig. 2:**
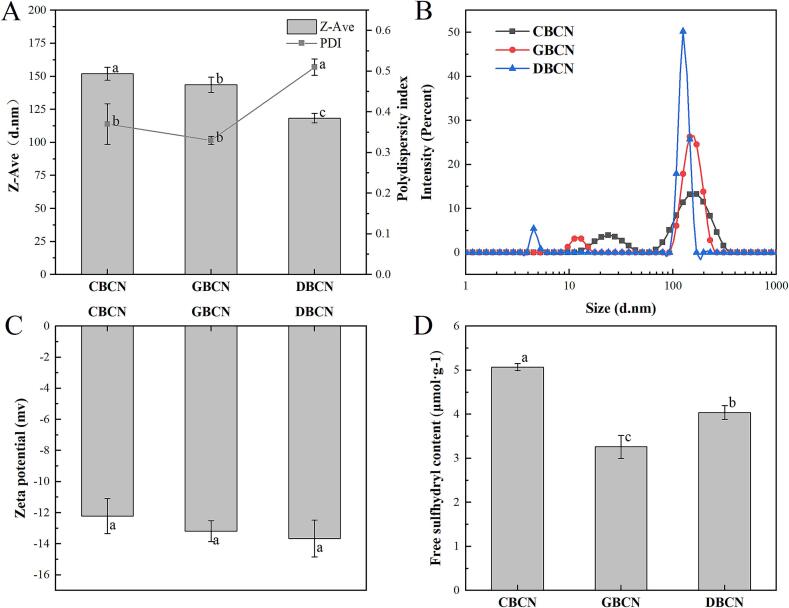
Average particle size and PDI (A), particle size distributions (B), zeta potential (C), and free sulfhydryl content (D) of β-casein.

The average particle sizes of CBCN, GBCN, and DBCN were observed at 152.00 nm, 143.60 nm, and 118.39 nm, respectively (*P* < 0.05), with all PDI values remaining below 0.6 (Fig. 2A). PDI values between 0.05 and 0.6 indicate a uniform particle size dispersion of the protein solution. The particle size of β-casein monomers derived from donkey milk was approximately 4.5 nm, which is significantly smaller than that of goat (∼11.23 nm) and cow (∼23.88 nm) milk (Fig. 2B). This is consistent with the hydrodynamic diameter of cow, goat, and donkey β-casein at 2 mg/mL at room temperature reported by Perinelli et al. (2019). Furthermore, the particle size of β-casein aggregates from donkey milk was around 125.6 nm, which is also significantly smaller than the corresponding sizes for goat (∼146.1 nm) and cow (∼169.9 nm) milk.

### 3.3 Zeta potential of β-casein

The zeta potentials for CBCN, GBCN, and DBCN were −12.22 mv, −13.18 mv, and −13.66 mv, respectively (Fig. 2C). Previous research has shown that solutions with higher absolute values of zeta potential possess stronger repulsive forces between molecules, thereby reducing the likelihood of aggregation and enhancing the stability of the solution system (L. Huang et al., 2022). Consequently, the stability of β-casein solutions was ranked in descending order as follows: donkey, goat, and cow milk.

### 3.4 Free sulfhydryl content of β-casein

The sulfhydryl group, a critical functional group in proteins known for its high biological activity, is capable of interconversion with disulfide bonds through the action of sulfhydryl/disulfide bond oxidoreductase. The concentrations of free sulfhydryl groups in the three β-casein proteins were found to be highest in cow milk, followed by donkey and goat milk, respectively (*P* < 0.05). This indicates that the β-casein from cow milk has a greater number of exposed sulfhydryl groups distributed on the surface of the protein molecules. There is a close relationship between sulfhydryl content and antioxidants, which provides support for the differences in the antioxidant properties of β-casein from the three milk sources observed in this study.

### 3.5 Secondary structure of β-casein

**Fig. 3:**
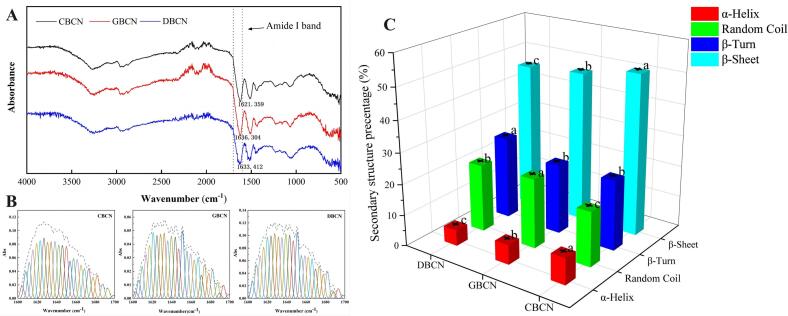
Amide Ι band (A), fitted graph (B), and secondary structure (C) of β-casein.

The vibrational frequency of the amide I band (1600–1700 cm^−1^) in the infrared spectrum depends on the hydrogen bonding characteristics between the carbonyl (C=O) and amine (N—H) groups. The absorption peaks within this band primarily reflect the secondary structures that are established both between and within the protein molecules. The characteristic absorption peaks of the β-casein amide I bands for cow, goat, and donkey milk were observed at the wavelengths of 1621.36, 1636.30, and 1633.41 cm^−1^, respectively (Fig. 3A). A correlation coefficient (R^2^ ≥ 0.99) between the fitted (Fig. 3B) and original plots. α-Helices and β-sheets in protein molecules are capable of forming compact, cavity-free structures, while the conformational stability and compactness of the irregularly coiled structures are comparatively inferior (M. Li et al., 2020). The elevated proportions of β-turns and random coils in the secondary structure of proteins, along with a reduced presence of highly ordered β-sheets, suggest a loose configuration. Specifically, the content of random coils in goat milk β-casein was 22.33 %, which is significantly greater (*P* < 0.05) than that of donkey and cow milk, at 21.72 % and 17.53 %, respectively (Fig. 3C). This aligns with prior research indicating a higher prevalence of irregular curls in β-CN from goat milk compared to that from cow milk (M. Li et al., 2020). Furthermore, the β-turn content of donkey milk β-casein was measured at 26.39 %, significantly surpassing that of goat and cow milk, at 22.31 % and 22.43 %, respectively (*P* < 0.05). Conversely, the β-sheet and α-helix content in cow milk β-casein was higher than that in donkey and goat milk (*P* < 0.05). Consequently, the reduced level of α-helixes and β-sheets, coupled with the increased presence of random coil structures in the β-casein of goat and donkey milk, contribute to a greater degree of molecular disorder and a more relaxed and open structural configuration. Thus, donkey and goat milk are more easily broken down during digestion compared to cow milk.

### 3.6 Turbidity and solubility of β-casein

**Fig. 4:**
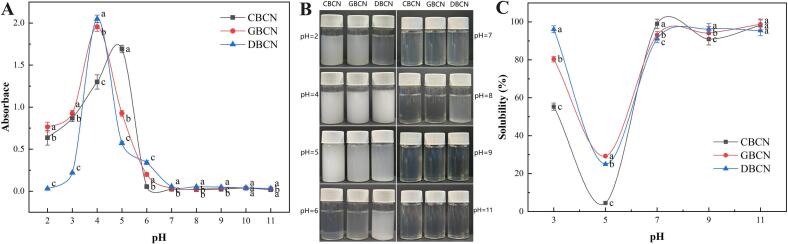
Turbidity (A), turbidity appearance (B), and solubility (C) of β-casein.

The turbidity values (Fig. 4A) and visual characteristics (Fig. 4B) of the three β-casein solutions at various pH levels show that all solutions exhibit peak turbidity at their isoelectric points. At pH 5, the turbidity of the β-CN from cow milk was significantly higher than that of goat and donkey milk, while at pH 4, the turbidity of the β-CN solution from goat and donkey milk surpassed that of cow milk. Previous studies have determined the isoelectric point of cow milk β-CN to be close to 4.6, while that of goat milk is around 4.1 (Recio et al., 1997). Thus, the isoelectric point for donkey milk β-CN is likely to be close to 4. The isoelectric point of donkey β-CN is reported to be more acidic than that of cow β-CN (Zhang, Polidori, et al., 2024). Turbidity levels were found to be higher in acidic conditions compared to alkaline conditions. However, at pH 2, the turbidity of the donkey milk β-casein solution was significantly lower than that of both cow and goat milk.

Solubility is an important physical, chemical, and functional property of proteins. It can reflect the degree of denaturation and aggregation of the internal structure of proteins. The solubility curves of the three β-caseins at different pH values are shown in Fig. 4C. The solubility of the three β-caseins has obvious pH dependence, with all solubility curves decreasing near the isoelectric point and increasing far away from it. This is because the pH of the protein solution is near the isoelectric point and the positive and negative charges on the surface of the protein molecules are equal, which enhances the interaction force between the protein molecules and other molecules (Post et al., 2012). Table 1 shows that the surface of the cow β-CN molecule contains more hydrophobic groups than goat and donkey, which undergo aggregation through hydrophobic interactions, making cow β-CN less soluble than that of goat and donkey. When the pH of the solution is far from the isoelectric point, the protein molecules are in an acidic or alkaline environment. At this time, the protein molecules have an electrostatic charge. Due to electrostatic repulsion and ionic hydration, it is not easy for the protein molecules to aggregate with each other and the solubility is therefore higher. Moreover, the ionic strength of the solution is increased to build up the protein surface charge and promote the unfolding of the protein structure. At this time, the cow β-CN solubility was lower than that of sheep and donkey milk. This was caused by the weaker electrostatic repulsion of cow β-CN and its more compact secondary structure with higher sulfhydryl content, leading to less unfolding for the spatial structure.

**Table 1:** Surface hydrophobicity, emulsification properties, and foaming characteristics of β-casein derived from cow, goat, and donkey milk.

Sample	CBCN	GBCN	DBCN
Surface hydrophobicity	386.78 ± 12.08^a^	287.84 ± 12.34^c^	295.73 ± 15.02^b^
EAI (m^2^/g)	68.17 ± 1.59^b^	65.14 ± 1.31^c^	79.22 ± 1.74^a^
ESI (%)	37.63 ± 1.86^b^	37.90 ± 2.41^b^	43.14 ± 2.25^a^
FC (%)	28 ± 1.5^b^	20 ± 1.5^c^	32 ± 1.1^a^
FS (%)	28.57 ± 2.1^b^	10 ± 1.8^c^	37.5 ± 2.4^a^

Note: All measurements were performed in triplicate on three samples. a-c Different letters indicate significant differences between means (*p* < 0.05).

### 3.7 Surface hydrophobicity, emulsification properties, and foaming characteristics

The degree of surface hydrophobicity in a protein solution is directly correlated with the concentration of hydrophobic amino acid residues. Table 1 reports the surface hydrophobicity of β-casein solution from the three different milk sources. The surface hydrophobicity of CBCN is significantly greater than that of DBCN and GBCN. This observation aligns with the previous literature, which indicates that the surface hydrophobicity of cow β-CN exceeds that of donkey β-CN at a pH of 7 (Zhang, Vincenzetti, et al., 2024). Moreover, the emulsification and stability of DBCN were significantly higher than those of GBCN and CBCN (Table 1). This is because the hydrophobicity of the protein surface influences the emulsification. More hydrophobic residues result in more hydrophobic properties at the protein surface, which is closer to the oil phase. The interfacial tension between oil and water is reduced and a film is formed, which promotes emulsification (Tigges et al., 2010). In addition, small protein particles are conducive to the rapid diffusion of protein in the emulsion, which accelerates the aggregation of protein to the interface, reduces the surface tension, and improves the emulsification and emulsification stability of the emulsion (S. Wang et al., 2021). DBCN has higher surface hydrophobicity than GBCN and the smallest particle size, and thus its emulsification property is optimal among the three sources. This is followed by CBCN, with the highest surface hydrophobicity. The foamability and stability of DBCN were significantly higher than those of CBCN and GBCN, with the latter exhibiting the lowest values (Table 1). This is because more hydrophobic residues promote the formation of a gas–liquid interface, enhance its surface activity, and improve protein foamability (Delahaije & Wierenga, 2022). In addition, the decrease in particle size is associated with an increase in FC (Morales et al., 2015). The particle size of donkey β-casein molecules is smaller compared to that of the other two sources, the absolute values of potential is large, the aggregation strength is relatively weak, the mobility of protein molecules adsorbed to the gas–liquid interface is enhanced, and the foaming ability of donkey β-casein is increased. The poor FS of GBCN is attributed to its low hydrophobicity, which affects foam capacity at a given concentration in a protein-poor state (Delahaije & Wierenga, 2022). For many dairy products, such as beverages, cakes, etc., where emulsification and foaming are more important, donkey β-CN will meet the overall product quality and consumer acceptance requirements.

### 3.8 Antioxidant capacity of β-casein

**Fig. 5:**
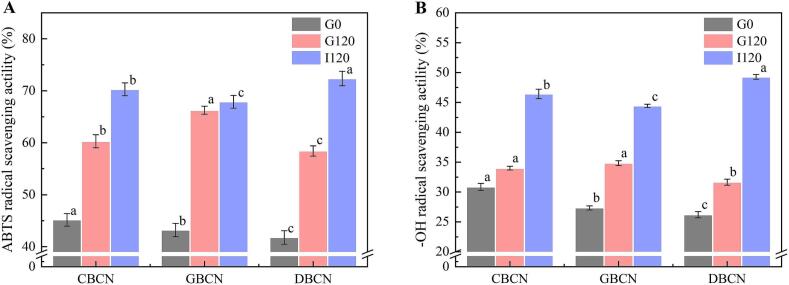
ABTS (A) and -OH (B) radical scavenging activity of β-casein.

Fig. 5 reports the antioxidant capacity of the β-casein solutions and their corresponding digestive juices. The clearance rates of the ABTS and -OH free radicals in CBCN were significantly higher than those observed in GBCN and DBCN. This shows that the β-casein derived from cow milk exhibits the highest antioxidant capacity among the three sources, while that of donkey milk is the lowest. The superior antioxidant properties of cow β-casein can be attributed to the greater number of exposed sulfhydryl groups present on its molecular surface (Balendiran et al., 2004). Following digestion, all three β-casein digests showed varying degrees of enhancement in their ability to scavenge •OH and ABTS free radicals. This is likely due to the formation of antioxidant peptides during the digestion process (Bucevic-Popovic et al., 2014; L. Li et al., 2018). The antioxidant properties of GBCN were found to be inferior to those of CBCN, which is consistent with the literature regarding casein digests from goat and cow sources (Assem et al., 2018). Notably, the free radical scavenging capacity of DBCN significantly enhanced post-digestion, corroborating previous research that indicates a stronger antioxidant capacity in donkey milk digests (L. Li et al., 2018). In terms of •OH and ABTS radical scavenging rates, the antioxidant capacity of β-casein digests from the three milk sources ranked as follows: DBCN, CBCN, and GBCN. This further suggests that digesting donkey β-casein yields a greater quantity and potency of antioxidant peptides, resulting in significantly enhanced antioxidant properties.

### 3.9 Hydrolysis degree

**Fig. 6:**
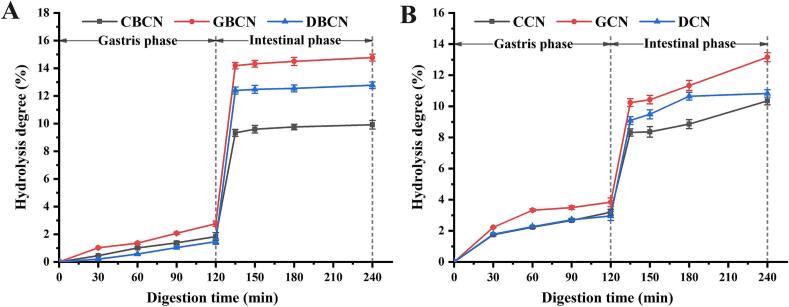
Hydrolysis degree of β-casein (A) and casein (B).

The digestibility of protein can be assessed by quantifying the degree of hydrolysis (DH) of the protein. In order to study the digestion of β-casein in a complex protein system such as infant formula. Therefore a comparison was made between the speed of digestion of a complex casein system and a single β-casein system. The relationship between digestion and β-casein type and content was explored. Fig. 6A and B present the DH of β-casein and casein derived from the three dairy sources during a simulated infant digestion process. An extension of hydrolysis duration correlates with an increase in the degree of protein hydrolysis, which eventually tends to stabilize due to a reduction in available hydrolysis sites. During the gastric digestion phase, the DH of the three milk sources exhibits a modest increase, albeit at relatively low levels. This can be attributed to the formation of large and numerous protein aggregates at a pH of 4. Notably, the increment in DH during the gastric phase for the β-casein group (Fig. 6A) was significantly lower than that observed in the casein group from the same milk source (Fig. 6B). This discrepancy arises from the larger size of the flocculated samples in the β-casein group compared to those in the casein group (Moitzi et al., 2008). As can be seen from Fig. 6A, the various samples within the β-casein group exhibited differing hydrolysis conditions during the gastric digestion stage, indicating that varying degrees of hydrolysis occurred among the samples from different milk sources. The DH of GBCN was found to be higher, suggesting that its enhanced gastric digestibility may be linked to its small particle size and the formation of diminutive particles resulting from weak agglutination in the stomach. This is consistent with previous literature that reports casein from goat to be more digestible than that from cow (Hodgkinson et al., 2018). Casein tends to be hydrolyzed more during the intestinal phase compared to the gastric phase. The DH of the three β-casein group samples also exhibited slight variations, with the DH increments of GBCN (14.78 % ± 0.25 %) and DBCN (12.78 % ± 0.24 %) at post-intestinal digestion determined as significantly greater than that of CBCN (9.92 % ± 0.31 %). Thus, β-CN from goat and donkey milk demonstrates superior digestibility compared to cow milk, thereby facilitating its absorption and utilization by the human body. However, the casein group contained indigestible α_s_-casein, which had a significantly lower DH than the β-casein group from the same milk source (Lang et al., 2021). In summary, the ethnic origin and content of β-casein significantly influence the digestion and hydrolysis processes in both the stomach and intestine.

### 3.10 Microstructure

**Fig. 7:**
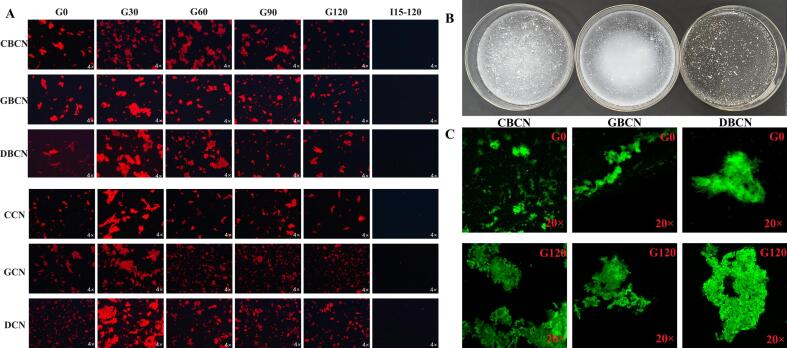
Changes in the appearance and structure of casein and β-casein during digestion. (A) Micrographs (4 × CLSM) of gastric digest (G) after 0 min (G0), 30 min (G30), 60 min (G60), 90 min (G90), and 120 min (G120) and intestinal digest (I) after 15 min (I15) and 120 min (I120). (B) Appearance of CBCN, GBCN, and DBCN. (C) Micrographs (20 × CLSM) of gastric digest (G) after 0 min (G0) and intestinal digest (I) after 120 min (I120).

Fig. 7A presents the microstructural characteristics of β-casein and casein digests derived from the three distinct milk sources. G0 indicates the state whereby the sample pH was adjusted to 4 and no enzyme was added. Notably, both the β-casein and casein samples exhibited significant aggregation, resulting in extensive flocculation at this pH level. This can be attributed to the reduction in pH and the enzymatic hydrolysis of proteins, which markedly diminishes the spatial stability and charge of the protein molecules, thereby facilitating the formation of a protein network structure (Markoska et al., 2021). Furthermore, the flocculation size observed in the β-casein samples was greater than that of the casein samples from the same milk source. This disparity may be related to the chaperone-like properties of β-CN, which not only contributes to the formation of casein micelles but also facilitates the creation of oligomeric micelles through hydrophobic interactions (Portnaya et al., 2008). Fig. 7A depicts the changes in the digested microstructure of the three β-caseins. Specifically, the clots formed by CBCN and DBCN during the gastric phase were significantly larger than those produced by GBCN. This may be due to the enhanced hydrophobic interactions present in CBCN and DBCN, which strengthen aggregation and facilitate the formation of larger aggregates, thereby impeding further hydrolysis by pepsin and resulting in larger clots. As the gastric phase progressed and the pH of the system gradually increased, the structure of the protein flocculation became increasingly loose, facilitating enzymatic hydrolysis. Consequently, the number of clots within the system was significantly reduced. However, the β-casein samples retained a greater quantity of larger protein aggregates compared to the casein group. Thus, it can be concluded that the ethnic source and content of β-casein significantly influence protein aggregation during the digestive phase, which has an impact on protein digestion. The appearance of the three β-caseins at the end of gastric digestion is shown in Fig. 7B. GBCN is the most fine-grained, followed by DBCN, and CBCN is the largest. Besides, as observed in the 20× microdetailed image in Fig. 7C, at the end of gastric digestion, the three β-caseins underwent structural splitting after hydrolysis by pepsin, with the periphery changing from smooth to irregularly toothed. The internal structure underwent an obvious collapse. Among them, the protein size of GBCN was significantly smaller than that of DBCN and CBCN, and its structure underwent a more pronounced collapse, becoming looser and more fully digested by the enzyme. The above results suggest that goat β-casein is more readily digested and absorbed than cow and donkey β-casein in simulated in vitro infant digestion.

## 4 Conclusion

In this study, liquid chromatography and amino acid sequence analysis yielded that goat and donkey β-CN were not cow A1 type β-CN, which could avoid the detrimental effects of digestion caused by BCM-7. Goat and donkey β-CN are less likely to aggregate in aqueous solution and have a more stable protein system because of their smaller particle size, larger absolute values of zeta potential and lower hydrophobicity than cow β-CN. The secondary structures showed that goat and donkey β-CN had more irregular curls and β-turns and were more loosely structured. All three β-CN showed good solubility away from the isoelectric point. The antioxidant properties of donkey β-CN digestion products were significantly better than those of goat and cow. Goat β-CN underwent more extensive hydrolysis and less protein flocculation in the static infant in vitro digestion model, indicating a more efficient digestion process. Therefore, the β-CN from goats and donkeys presents considerable potential as a viable alternative to cow β-CN for the formulation of infant nutrition products.

## CRediT authorship contribution statement

**Guiqin Liu:** Resources. **Ning Wang:** Writing – original draft, Data curation. **Xue Chen:** Validation. **Yaqian Jin:** Methodology. **Junnan Wan:** Formal analysis. **Yanhao Zhao:** Formal analysis. **Yiting Zhao:** Visualization, Supervision. **Cunfang Wang:** Project administration, Funding acquisition.

## Declaration of competing interest

The authors declare that they have no known competing financial interests or personal relationships that could have appeared to influence the work reported in this paper.

## Data Availability

Data will be made available on request.
